# The Oxygen Release Instrument: Space Mission Reactive Oxygen Species Measurements for Habitability Characterization, Biosignature Preservation Potential Assessment, and Evaluation of Human Health Hazards

**DOI:** 10.3390/life9030070

**Published:** 2019-08-27

**Authors:** Christos D. Georgiou, Christopher P. McKay, Richard C. Quinn, Electra Kalaitzopoulou, Polyxeni Papadea, Marianna Skipitari

**Affiliations:** 1 Department of Biology, University of Patras 26504, Greece; 2 NASA Ames Research Center, Moffett Field, CA 94035, USA; 3 SETI Institute, Carl Sagan Center, Mountain View, CA 94043, USA

**Keywords:** planetary oxygen-based reactive oxidants, instrument, habitability, biosignatures

## Abstract

We describe the design of an instrument, the OxR (for Oxygen Release), for the enzymatically specific and non-enzymatic detection and quantification of the reactive oxidant species (ROS), superoxide radicals (O_2_^•−^), and peroxides (O_2_^2−^, e.g., H_2_O_2_) on the surface of Mars and Moon. The OxR instrument is designed to characterize planetary habitability, evaluate human health hazards, and identify sites with high biosignature preservation potential. The instrument can also be used for missions to the icy satellites of Saturn’s Titan and Enceladus, and Jupiter’s Europa. The principle of the OxR instrument is based on the conversion of (i) O_2_^•−^ to O_2_ via its enzymatic dismutation (which also releases H_2_O_2_), and of (ii) H_2_O_2_ (free or released by the hydrolysis of peroxides and by the dismutation of O_2_^•−^) to O_2_ via enzymatic decomposition. At stages i and ii, released O_2_ is quantitatively detected by an O_2_ sensor and stoichiometrically converted to moles of O_2_^•−^ and H_2_O_2_. A non-enzymatic alternative approach is also designed. These methods serve as the design basis for the construction of a new small-footprint instrument for specific oxidant detection. The minimum detection limit of the OxR instrument for O_2_^•−^ and O_2_^2−^ in Mars, Lunar, and Titan regolith, and in Europa and Enceladus ice is projected to be 10 ppb. The methodology of the OxR instrument can be rapidly advanced to flight readiness by leveraging the Phoenix Wet Chemical Laboratory, or microfluidic sample processing technologies.

## 1. Introduction

On Earth, the production of reactive oxygen species (ROS) in soils is typically associated with the relatively high abundance of O_2_(g) in the atmosphere [1]. In other solar system environments, or space environments beyond our solar system, where O_2_(g) exists only in trace amounts (e.g., Mars [2], the Earth’s Moon [3,4], Europa [5], Saturn’s rings [6], interstellar clouds [7]) the production and accumulation of ROS is not precluded.

Even in planetary environments lacking O_2_(g), ROS can be produced by many well-known natural processes, for example, environments containing H_2_O, CO, and/or CO_2_ [8]. On the Moon (and presumably on Mars), ROS can be generated by the interaction of H_2_O ice with cosmic rays [9]. Experiments indicate that Lunar (and presumably Martian) dust can generate hydroxyl free radicals (^•^OH) via the Fenton reaction as demonstrated with Lunar simulants [10] and Fe-rich silicate minerals [11]. Freshly fractured Lunar regolith can produce large amounts of H_2_O_2_ and other ROS [12], which are considered to play a role in Lunar dust toxicity [13]. Although, none of the curated Apollo mission Lunar samples exist in a state that fully preserves the reactive chemical surfaces aspects (i.e., ROS) that would be expected to be present on the lunar surface, freshly ground Lunar soil has been shown to produce ^•^OH upon contact with H_2_O [14]. On Mars, reactive O_2_^+^ and O_2_^−^ can form through the release of reactive oxygen via scattering of CO_2_ ions from solid surfaces; where oxygen produced is preferentially ionized by charge transfer from the surface over the predominant atomic oxygen product [8]. ROS may also be produced by Martian regolith via silicate abrasion during dust storms [15] (e.g., by mechano-radical production [16]). Silica fracturing is known to generate surface free radicals (homolytic and heterolytic fracturing form Si^•^/SiO^•^, and Si^+^/SiO^−^, respectively), which upon reaction with H_2_O or H_2_O_2_ generate ^•^OH [17]. Such dust/silica-induced radicals may pose a serious human health hazard (verified by toxicity studies on mammalian cells [18]), during future manned missions to Mars and Moon.

Beyond Mars and the Moon, complex interactions between Saturn and its satellites Titan and Enceladus can cause the generation and movement of oxygen from the latter to the former [19,20]. Ice water from Enceladus south polar plumes can be radiolytically oxidized to H_2_O_2_ and O_2_, by energetic particles from Saturn’s radiation belts (mostly electrons). Such ROS emanating from this radiolytic gas-driven cryovolcanism can be continuously accumulated deep in icy regolith [19]. Concurrently, H_2_O molecules escaping from Enceladus’ plumes should be split by magnetospheric plasma (protons, H^+^_2_, water group ions) into neutral and charged particles (O^+^), which can enter Titan’s atmosphere and be captured by fullerenes (a hollow carbon atom shell, e.g., of C_60_). Exogenic keV O^+^ could become free oxygen within those fullerene aerosols, and eventually, fall free onto Titan’s surface. Such a process could be driven by cosmic ray interactions with aerosols at all heights, and can eventually, drive pre-biotic chemistry [20]. It has been suggested that ice-covered worlds require an external source of oxidants to maintain biological viability [21]. Hand et al. 2007, have proposed that oxidants produced by UV and ionizing radiation on the surface of icy worlds, such as Europa, can be carried down to the water column to react with reduced species to provide a source of redox energy [22].

In light of all these considerations, measurement of planetary ROS is of great interest for astrobiology, including the exploration of Titan, Enceladus, and Europa, and important for human missions to the Moon and Mars. However, instruments for the in situ specific detection of the key ROS O_2_^•−^ and O_2_^2−^ (e.g., H_2_O_2_) in these extreme environments have not yet been developed.

The justification for this type of instrument is supported by the results of the Viking Mars mission. In 1976, the Viking Lander performed biological experiments designed to detect extant life in Martian regolith. The reactivity of the Martian regolith was first indicated by the release of O_2_ in the Gas Exchange Experiment (GEX), and the decomposition of organics, contained a culture media, in the Labeled Release (LR) experiment [23,24,25]. In the GEX, up to ~770 nmoles O_2_(g) was produced from regolith samples (1 cm^-3^) upon humidification or wetting. The persistence of O_2_(g) release from samples that were heated to 145 °C for 3 h and then cooled prior to wetting or humidification, ruled out a biological explanation of the GEX results [23,26]. In the Viking LR, up to ~30 nmoles ^14^C labeled gas, presumed to be CO_2_, was released after regolith samples (0.5 cm^-3^) were wetted with an aqueous solution containing ^14^C-labeled organics [27,28]. The release of ^14^C-labeled gas in the LR was eliminated by heating the sample to 160 °C for 3 h and then cooling prior to the addition of the labeled aqueous organics. These results lead to the conclusion that the Martian surface material contains more than one type of reactive oxidants [23]. Metal salts of O_2_^•−^ were among the earliest proposed explanations for the thermally stable agent responsible for O_2_(g) release in the GEX. In the case of the LR, peroxide was among the earliest explanations proposed for the thermally liable agent responsible for ^14^CO_2_ release. In addition to the possible presence of metal salts of O_2_^•−^, it has been proposed that O_2_^•−^ is generated on Martian dust and regolith surfaces by a UV-induced mechanism [29]. Such a mechanism for O_2_^•−^ photo-generation has also been shown with Mars analog Mojave and Atacama regolith [1].

More recently, high levels of regolith perchlorate (ClO_4_^−^) were directly detected at the Phoenix landing site [30]. Following up on this result, the presence of ClO_4_^−^ at the Viking landing sites was inferred [31], and its presence at the Martian equator verified by the Sample Analysis at Mars (SAM) instrument on Mars Science Laboratory (MSL)-based on thermal analyses [32]. While the stability of ClO_4_^−^ under the conditions of the GEX and LR preclude it as a direct explanation for these experiments, it has been suggested that ClO_4_^−^ radiolysis products reproduce the major aspects of both experiments [25]. The form of the trapped O_2_, in particular, derived from ClO_4_^−^ radiolysis, was not identified, and it has been suggested that some fraction may exist as superoxide radical or peroxide [25,33]. This suggestion was confirmed by the finding that both of these oxidants are generated—together with ^•^OH—by *γ*-ray exposure of ClO_4_^−^ (mixed in Mars salt analogs) upon water wetting [34]. Preceding this finding, other possible oxidants present on the surface of Mars have been reviewed in detail [35]. In the context of instrument development for in situ analysis, it is useful to note that the expected concentration of oxidants, as inferred from the Viking Biology Experiments, is at the parts per million level (Table 1 in [36]).

Given the poorly understood nature and distributions of oxidants in Martian and Lunar regolith, there is a need for the development of flight instruments for their specific identification and quantification. The only flight instrument previously built for the quantitative, although non-specific, in situ determination of oxidants was the Mars Oxidant Experiment (MOX) instrument [36] as the United States contribution to the failed Soviet Union’s Mars ’96 mission. That instrument would have exposed materials-sensors to the Martian regolith and monitored their reaction with oxidants over time. Materials included various metallic (e.g., Al, Ag, Pb, Au) and organic layers (e.g., L- and D-cysteine).

New developments have made possible the detection of reactive oxidants, such as peroxides (O_2_^2−^), O_2_^•−^, and ^•^OH [1,34]. In planetary and terrestrial regolith, O_2_^•−^ may exist as adsorbed (O_2_^•−^_ads_) [1] or present in metal salts (Me^+^ O_2_^•−^), such as KO_2_ and NaO_2_ [37], and in ionic complexes with metals (Me^n+^− O_2_^•−^) of certain minerals and oxides [38,39]. Metal peroxides can exist as salts of metals with O_2_^2^_¯_ bonding either as Me^2+^O_2_^2−^ (e.g., CaO_2_, MgO_2_) or as Me^+^_2_O_2_^2−^ (e.g., Na_2_O_2_, K_2_O_2_). Metal peroxides can also exist as hydroperoxides (MeO_2_H; e.g., of Ti^4+^, Zr^4+^, and Ce^4+^) [40]. The presence of Mg^2+^, Ca^2+^, K^+^, and Na^+^ ions on Martian regolith (measured with the Phoenix Mars Lander Wet Chemistry Lab [30,41,42]) and on Lunar regolith [43], may provide the needed counter ions for stabilization of metal salts of O_2_^•−^, peroxides, and hydroperoxides in the regolith. Metal salts of O_2_^•−^ and hydro/peroxides (i.e., O_2_^2−^) can undergo aqueous decomposition at neutral pH, releasing O_2_ and H_2_O_2_ (Table 1).

## 2. Principle of Operation of the OxR (for Oxygen Release) Instrument

The OxR instrument for the detection and quantification of O_2_^•−^ and O_2_^2−^ addresses priorities for human exploration of Mars and the Moon as highlighted in the NASA plan to “Explore Moon to Mars” which will use the Moon as “a testbed for Mars […] and beyond.” [45].

Our approach is to quantitatively convert peroxides and superoxide radicals into O_2_(g), which can then be detected easily, precisely, and with very high sensitivity. The principle of the OxR instrument is based on the enzymatic conversion of the dismutation and hydrolysis products of superoxide radicals (O_2_^•−^ adsorbed on mineral surfaces, O_2_^•−^_ads_, or released by the dissociation of metal salts of O_2_^•−^) and peroxide (O_2_^2−^ as H_2_O_2_ or released by the hydrolysis of metal peroxides) to O_2_, followed by quantitative detection using an O_2_ electrode.

The OxR instrument design includes a sealable, temperature- and pressure-controlled sample chamber. The chamber is equipped with an O_2_-sensor, and inlets for the sequential dispensing of three reagents, after each of which the concentration of released O_2_ is measured. The two enzymatic reagents (Cu/Zn-superoxide dismutase, SOD, and catalase, CAT) used are stored in a solid form separated from their aqueous solvents (to withstand cosmic radiation exposure). The third reagent, acetonitrile (ACN), is separately stored at any temperature above its melting point (−46°C). The enzymic reagents are mixed with their solvents right before use either by storing them in separate reagent crucibles (analogous to those used in the Wet Chemistry Laboratory of the 2007 Phoenix Mars Scout Lander mission), or in (commercially available) dual-chamber pre-fillable syringes (one chamber for storing the enzyme reagent in solid form, and the other for its solvent, to be mixed upon piston movement), and their sequential dispensing in the chamber. The OxR instrument can detect released O_2_ by electrochemical or solid state optical O_2_-sensing electrodes. Both electrode types are commercially available. Optical O_2_-sensing electrodes are based on the luminescence quenching by O_2_, and are sensitive enough to measure O_2_ at ~1 nmole O_2_ per cubic cm (cm^−3^) of regolith or water, i.e., much lower than that detected by the GEX (775 nmoles cm^-3^ regolith). This translates to a minimum instrument detection limit for metal salts of O_2_^•−^ and O_2_^2−^ of 0.01 ppm (= 10 ppb) for Martian and Lunar regolith or O_2_^2−^ in Europa and Enceladus water. This sensitivity corresponds to ~10 µg O_2_^•−^/O_2_^2−^ kg^−1^ Mars or Lunar surface regolith (based on a density of 1.4–1.6 g cm^−3^ for Mars [46,47], and for the Moon [48]).

## 3. Enzyme-Based ROS Specificity of the OxR Instrument

We have developed an enzymatic methodology (OxR assay) for the detection of total O_2_^•−^ (the sum of O_2_^•−^_ads_, Me^+^ O_2_^•−^, and Me^n+^− O_2_^•−^) and total O_2_^2−^ (the sum of Me^2+^O_2_^2−^, Me^+^_2_O_2_^2−^, and MeO_2_H), for terrestrial field and planetary applications [49]. The use of enzymes for the OxR assay provides specificity and quantification of regolith O_2_^•−^ (reactions 1–3) and O_2_^2−^ (reactions 4–6) through the measurement of the O_2_ that is enzymatically released during dismutation/hydrolysis. Specifically, this is achieved using the following enzymatic reactions [44]: (i) the SOD-catalyzed dismutation of 1 mole O_2_^•−^ to ½ mole O_2_ and ½ mole H_2_O_2_, and (ii) the CAT-decomposition of 1 mole H_2_O_2_ (from dismutated O_2_^•−^ and hydrolyzed metal peroxides/hydroperoxides) to ½ mole O_2_.

The enzymatic reaction steps of the OxR assay have been established by the following experimental testing [49]: (i) The effective scavenging of O_2_^•−^ via dismutation to O_2_ and H_2_O_2_ by SOD; (ii) the decomposition of H_2_O_2_ (from the hydrolysis of peroxides and the dismutation of O_2_^•−^) to O_2_ (by CAT) in the presence of ClO_4_^−^ and carbonate (CO^2−^; both Martian regolith constituents), and in potassium phosphate plus diethylene–triamine–pentaacetic acid (DTPA) buffer (pH 7.2). Phosphate is an H_2_O_2_-stabilizer [50], and DTPA acts as chelator of any soluble transition metal ions, which can destroy H_2_O_2_ via the Fenton reaction [51] and O_2_^•−^ via oxidation to O_2_); (iii) the functional stability of the OxR assay enzymes SOD and CAT to cosmic rays upon exposure to *γ*-radiation; (iv) the simulation of the OxR assay by indirect testing on commercial analogues of metal salts of O_2_^•−^ and O_2_^2−^, and directly on O_2_^•−^ and H_2_O_2_. The enzymatic (and accompanying non-enzymatic) reactions involved in the OxR assay are presented in Table 2.

## 4. OxR Assay Simulation Verification on Mars-Analog Regolith

The OxR assay was performed using a semi-sealed liquid-phase O_2_ electrode with known concentrations of O_2_^•−^ and H_2_O_2_, and in the presence/absence of Mars-like regolith from the Mojave (CIMA volcanic field) and the Atacama deserts. The assay was further validated on commercial sources of metal salts of O_2_^•−^ (KO_2_) and O_2_^2−^ (Na_2_O_2_, CaO_2_, MgO_2_) in the presence of CO^2−^ and ClO_4_^−^ (both are present in Martian regolith). *Gamma*-radiation experiments were performed to evaluate the stability of the OxR assay enzymes CAT and SOD against cosmic radiation [49].

**Figure 1:**
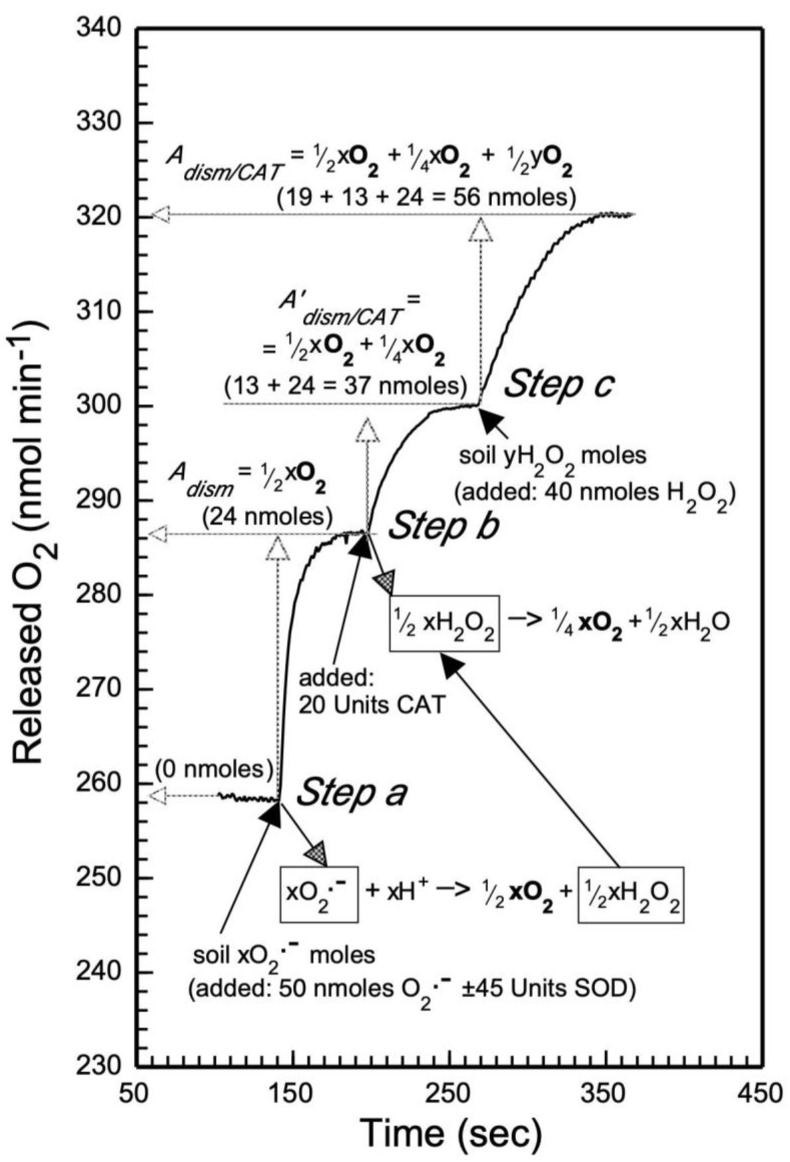
Simulation of the OxR (Oxygen Release) assay on O_2_^•−^ and H_2_O_2_: It is performed in the presence/absence of Mars-like regolith from Mojave and Atacama deserts with a liquid-phase Clark-type O_2_ electrode [49]. It is initiated (in *step a*) by the addition of 50 nmoles O_2_^•−^ (simulating regolith O_2_^•−^, represented as *x* O_2_^•−^ moles) in the absence or presence of 45 units Cu/Zn-superoxide dismutase (SOD), and the concentration of released O_2_ (by the SOD-catalyzed dismutation reaction of O_2_^•−^) is recorded (as reading *A_dism_* = ½*x*O_2_; see *Treatment A* in text), which is equal to the second dismutation reaction product H_2_O_2_ (= ½xH_2_O_2_). In a subsequent *step b*, the addition of catalase (CAT) causes the additional release of O_2_ (via the CAT-catalyzed decomposition of H_2_O_2_, the second product of O_2_^•−^ dismutation), which is also recorded (as reading *A_dism/CAT_* = ¼*x*O_2_ plus the already released ½*x*O_2_; see *Treatment B* in text). If there are also metal O_2_^2−^ or free H_2_O_2_ present in regolith (represented as *y*H_2_O_2_ moles), these are simulated in Figure 1 by the addition of 40 nmoles H_2_O_2_ (in *step c*). These peroxides will also be decomposed by the CAT (added in *step b*) to ½*y*O_2_, and in this case, the total released O_2_ will be recorded in *step c* as reading *A_dism/CAT_* (the sum ½*x*O_2_ + ¼*x*O_2_ + ½*y*O_2_).

The electrode reaction chamber was filled with 1 mL potassium (K)-phosphate-DTPA buffer (0.25 M K-phosphate buffer, pH 7.2, containing 10 mM DTPA) to which the assay reagents (O_2_^•−^, H_2_O_2_, SOD, and CAT) were added at constant room temperature (RT). As already noted, DTPA chelates any soluble transition metal ions that can destroy H_2_O_2_ and O_2_^•−^. Moreover, DTPA will also prevent such chelated metals from inactivating the OxR assay protein reagents SOD and CAT via their oxidation by ^•^OH (produced by way of the Fenton reaction) or via direct inhibition. The OxR assay was experimentally tested with known concentrations of O_2_^•−^ and H_2_O_2_ added in the Clark-type O_2_ electrode, as illustrated in Figure 1 [49]. To validate the OxR assay enzymatic reactions 1 and 7 (in Table 2) in the Clark-type O_2_ electrode chamber, the following treatments were performed (data are shown in Figure 1):

*Treatment A* (reaction 1, see Figure 1 *step a*): SOD-catalyzed dismutation of O_2_^•−^ to O_2_ and H_2_O_2_). Seventy microliters of O_2_^−^ stock solution was added (final 50 µM O_2_^•−^ or 50 nmoles) to the O_2_ electrode chamber, which contained 1 mL K-phosphate-DTPA buffer and ± 45 units (U) SOD (10 µl of a 4500 U ml^−1^ stock made in ddH_2_O), and the released O_2_ concentration was recorded until a plateau was reached.

*Treatment B* (reaction 7 in Table 2, see Figure 1 *step b*): CAT-catalyzed conversion to O_2_ of H_2_O_2_ released via SOD-catalyzed dismutation O_2_^•−^ derived by O_2_^•−^ hydrolysis, and H_2_O_2_ released from peroxides via hydrolysis. After measuring the 1st O_2_(g) plateau (*Treatment A*), 20 U ml^-1^ CAT (10 µl 2000 U ml^−1^ stock) was added to the resulting reaction mixture, and after the 2nd O_2_(g) plateau was reached, 10 µl 4 mM H_2_O_2_ (final 40 µM or 40 nmoles) was added, and the 3rd O_2_(g) plateau was recorded (Figure 1 *step c*).

*Mathematical treatment of the data derived from treatments A and B*: Assuming the presence of *x* O_2_^•−^ and *y*H_2_O_2_ moles in the K-phosphate-DTPA buffer in the Clark O_2_ electrode chamber, these supero/peroxidants were calculated from the experiments illustrated in Figure 1 as follows. The released O_2_ concentrations measured by the O_2_ electrode during *treatments A* and *B* (designated *A_dism_* and *A_dism/CAT_*, respectively) are described by the following molar equations and are based on the molar stoichiometry of the reactions 1 and 7 (in Table 2):

*A_dism_* = ½*x*O_2_;

simplified: *A_dism_* = ½*x*, where *x* is O_2_^•−^ moles

*A_dism/CAT_* = ½*x*O_2_ + ¼*x*O_2_ + ½*y*O_2_;

simplified: *A_dism/CAT_* = ¾*x* + ½*y*, where *y* is H_2_O_2_ moles

The molar concentrations of *x*O_2_^•−^ and *y*H_2_O_2_ are then estimated using the following mathematical equations, derived by appropriately combining the molar equations *A_dism_* and *A_dism/CAT_*:

O_2_^•−^ moles (= *x*) = 2*A_dism_*

H_2_O_2_ moles (= *y*) = 2*A_dism/CAT_* − 3*A_dism_*

The released O_2_ concentrations (*A_dism_* and *A_dism/CAT_*) during the OxR assay (shown in Figure 1) matched the concentrations predicted by the stoichiometry of each of the assay reactions 1 and 7. Indeed, when the values *A_dism_* (corresponding to 24 nmoles from *step a*) and *A_dism/CAT_* (corresponding to 37 nmoles from *step b*, or 56 nmoles from *steps b* plus *c*) are inserted to the above molar equations for O_2_^•−^ and H_2_O_2_, their calculated experimental concentrations are statistically equal to their concentrations which were added in the O_2_ electrode chamber.

*Simulation of cosmic radiation effect on the OxR assay enzymic reagents*: Another consideration for the OxR instrument is whether its enzymatic reagents SOD and CAT would be functional upon exposure to cosmic radiation levels expected during missions to Mars, the Moon, and possibly icy satellites Jupiter and Saturn. To address this question for Mars and Moon, cosmic radiation simulation experiments were performed [49], where solid SOD and CAT were exposed to *γ*-radiation at a dose range comparable to that which would be received during a space mission. Activities were also determined for these enzymes in various concentrations (% v/v) of ACN since 100% ACN is used to wet the regolith sample to quantitatively purge out any unknown source trapped O_2_. The SOD retained functional activity after exposure to a *γ*-radiation dose of 6 Gy (an equivalent to the cosmic radiation dose received from 38 round trips to Mars [53]). The CAT specific activity was unaffected up to ~3 Gy (equivalent to 19 round trips to Mars, and many more trips to the Moon) after which it decreased linearly to 40% (of its unexposed activity) at 6 Gy. SOD activity was unaffected in up to 50% ACN, while CAT activity decreased in a manner that matched the ACN concentration. For example, an initial CAT specific activity of ~3 U µg^−1^ at 0% ACN decreased by 50-fold at the maximum tested 50% ACN. This result indicates that for an OxR assay based on 3 U µg^−1^ CAT at 0% ACN in laboratory testing, the CAT concentration should be increased 50-fold (i.e., 150 U µg^−1^) plus a margin for flight, if a 50% ACN concentration is optimum for instrument implementation.

## 5. The Potential of the OxR Assay for a Field-Deployable Instrument

**Figure 2:**
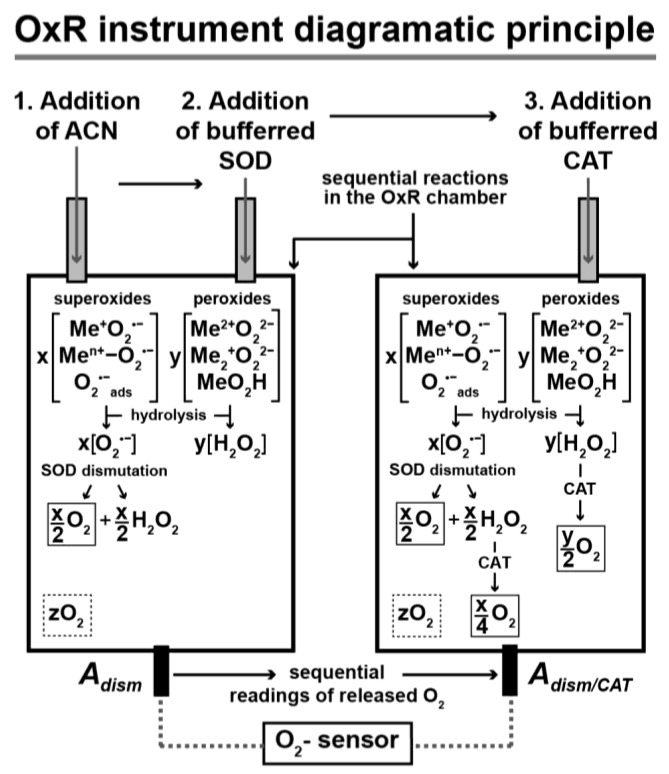
Diagrammatic principle of an OxR assay-based field instrument for the identification/quantification of regolith superoxide radicals and peroxides (shown as *x*O_2_^•−^ and *y*H_2_O_2_ moles, respectively): Regolith sample is subjected to the following released O_2_ recording procedures. In step 1, the regolith is wetted with anhydrous ACN to flush out loosely bound O_2_ (designated *z*O_2_ moles; enclosed in dotted squares) for (i) canceling out any background O_2_ and (ii) measuring it as coming from unidentified sources. In step 2, SOD is administered in the instrument chamber dissolved in K-phosphate-DTPA buffer (pH = 7.2) at an equal (at least) to ACN volume (resulting in at least 50% ACN), and the released O_2_ (enclosed in solid-line square, which results from the group of metal O_2_^•−^ via SOD-catalyzed dismutation of their hydrolysis product O_2_^•−^, together with H_2_O_2_) is recorded by the chamber O_2_ sensor as reading *A_dism_*. In a subsequent third step, K-phosphate-DTPA-buffered CAT is introduced in the same chamber, where the additional released O_2_ (from the decomposition of H_2_O_2_ coming from the hydrolysis of both groups of metal O_2_^•−^ and O_2_^2−^) is summed to that released from step 2 (and enclosed in three solid-line squares), and is recorded as reading *A_dism/CAT_*. The values of *A_dism_* and *A_dism/CAT_* (their net values determined by the experimental values designated by the arrows pointing at them on the Y-axis) are then used to determine the moles of regolith O_2_^•−^ and H_2_O_2_, using their respective equations: O_2_^•−^ = 2*A_dism_* (= x), and H_2_O_2_ = 2*A_dism/CAT_* - 3*A_dism_* (derived as shown in Section 4, ‘OxR assay simulation verification on Mars-analog regolith’).

The OxR assay can be extended to the search of possible metal supero/peroxidant cycles in terrestrial and extraterrestrial ecosystems. We expect that the full instrument can be packaged in 1 U (i.e., CubeSat sized at 10 cm/side) using a reaction chamber scheme with an O_2_-sensor (to monitor the enzymatic release of O_2_ from O_2_^•−^ and O_2_^2−^ in a regolith sample during interaction (under constant mixing) with SOD and CAT, as illustrated and described in Figure 2.

During operation, the first step of mixing the regolith with anhydrous ACN is very crucial for the following reasons: ACN (actually containing 0.2 mM dicyclohexano-18-crown-6 ether, CE) flushes loosely bound O_2_ from unknown sources (designated *z*O_2_) necessary for instrument calibration at the same time the CE component, will facilitate O_2_^•−^ dissociation from superoxo metal salts [1,54,55], and together they stabilize O_2_^•−^ for the subsequent enzymic steps 2 and 3 (Figure 2). In other words, the ACN-CE solvent used in step 1 prevents the dismutation of regolith O_2_^•−^ to O_2_ that would occur with the use of an aqueous solvent (see reaction 1 in Table 1), which would make the determination of background *z*O_2_ (and, thus, of *x* O_2_^•−^ and *y*H_2_O_2_ moles) impossible. It should also be noted that although the OxR assay can quantify O_2_ released from unknown sources (i.e., *z*O_2_), it cannot discriminate the H_2_O_2_ generated by the hydrolysis of metal superoxide radicals and peroxides from that of any free H_2_O_2_ (possibly existing in mineral pore spaces). The accurate quantification of metal superoxide radicals and peroxides by the OxR requires that their initial hydrolysis products O_2_^•−^ and H_2_O_2_, respectively, remain stable for SOD and CAT treatment. It has been already noted, the metal chelator DTPA and the phosphate buffer reagents will scavenge inorganic cations that affect the stability of O_2_^•−^ and H_2_O_2_. Even if a fraction of H_2_O_2_ converts to O_2_ by factors other than CAT (e.g., by high CO^2−^ concentration and high regolith alkalinity [49], or by the catalysts MnO_2_ [56], or silver, platinum, lead, ruthenate, and RuO_2_, which decompose H_2_O_2_ to O_2_ in alkaline solution [57]), this will not affect the accurate determination of metal superoxide radicals and peroxide concentrations since these factors will complement the conversion of H_2_O_2_ to O_2_ by CAT.

*Non-enzymatic OxR instrumentversion*: We have also developed a non-enzymatic OxR assay for cases where enzymatic stability may be insufficient (e.g., missions to Titan, Europa, and Enceladus) or when the required long-term −20 °C SOD and CAT storage is not possible. Moreover, some future rovers may long outlast their expected life times (as past ones have done), and for whichever rover carries an OxR instrument the reagent enzymes may degrade over the years, whereas the inorganics may be more durable. A non-enzymatic version of the OxR instrument is based on the following reagents, which we have preliminarily tested successfully (data not shown):

In place of SOD, CuSO_4_, MnCl_2_, and MnSO_4_ can be used:

1. CuSO_4_ (at 0.1 to 300 µM) and MnCl_2_ (at 0.1 to 100 µM)) [58,59]; MnCl_2_ dismutates O_2_^•−^ as effectively as SOD does [59].

2. MnSO_4_ (at 0.1 mM) has a rate constant for O_2_^•−^ dismutation *k* = 2.3 × 10^6^ M^−1^ s^−1^ (in 5 mM HEPES, pH = 7.8) [52]. This is 10-fold higher than the rate constant for the spontaneous aqueous dismutation of O_2_^•−^ (2 O_2_^•−^ + 2 H_2_O → 2 OH^−^ + H_2_O_2_ + O_2_; *k* = 2x10^5^ M^−1^ s^−1^ at pH = 7.8 [60]).

In place of CAT, the following inorganic reagents can be used:

1. MnO_2_ acts as CAT-mimetic (2H_2_O_2_ → 2H_2_O + O_2_) [56].

2. Ferricyanide [Fe(CN)_6_^3−^; FECN]. FECN reacts with H_2_O_2_ at a different stoichiometry than that of its CAT-catalyzed decomposition [½yH_2_O_2_ + Fe(CN)_6_^3−^ → Fe(CN)_6_^4−^ + H^+^ + ½yO_2_ [61,62]]. However, the use of FECN modifies the set of the equations for the determination of O_2_^•−^ and H_2_O_2_ via released O_2_ (specifically the equation for H_2_O_2_). These are the following, designating as *A_dism/FECN_* as the reading value (by the O_2_-electrode) for the released O_2_ after treatment with FECN:

**O_2_**^•−^ moles = 2*A_dism_* (same as with the enzymatic version of the OxR instrument)

**H_2_O_2_** moles = A_dism/FECN_ − 2A_dism_

Concluding, the principle of the OxR assay can be used for the development of an instrument for the detection of planetary and terrestrial O_2_^•−^ and O_2_^2−^ with the following considerations:

1. OxR assay enzymes SOD and CAT are used in excess; they are sufficient when used even in the amount of a few activity units.

2. SOD and CAT are stored (below −20 °C for long-term storage) separate from their aqueous solvents, and are mixed right before administration. This can be accomplished by storing them, for example, in two separate reagent crucibles (analogous to those used in the WCL instrument of the 2007 Phoenix Mars Scout Lander mission [63]), or in (commercially available) dual-chamber pre-fillable syringes (one chamber for storing the enzyme and one for its solvent, to be mixed upon piston movement), followed by their sequential dispensing in the instrument’s regolith chamber (under continuous mixing of its reagents).

3. The instrument can use solid state electrochemical or optical sensing O_2_-electrodes of high sensitivity. There are commercially available O_2_ probes (e.g., sensor type PSt6, by PreSens Precision Sensing GmbH, Regensburg, Germany) that are based on the luminescence quenching by O_2_, and are sensitive enough to measure O_2_ at much lower concentrations (~1 nmole O_2_ cm^-3^ regolith) than that (775 nmoles) detected by the GEX [26]. For example, the typical detection limit of the PreSens sensor PSt6 is 0.002% O_2_, with 1 ppb and 0.5 ppm for aqueous and gaseous O_2_, respectively. The PreSens Precision Sensing O_2_ probes come either as needle-type optical fiber probes (with a tip size < 50 μm, protected, e.g., inside a stainless-steel needle), or as implantable probes (with a tip size of < 50 to 140 μm, while the outer diameter ranges from 140 μm to 900 μm). Therefore, O_2_ sensing by the OxR instrument with solid-state sensors can be done in both gaseous and liquid phase. Regarding released O_2_ partition between liquid and headspace in the sample chamber, underestimation of the released reactive O_2_ due to such exsolution can be addressed by either adding an extra gas phase O_2_ sensor, or by the calculation of the partition between liquid and gas phase at the set pressure and temperature.

4. Respective ACN and SOD reagent process steps 1 and 2 are omitted in testing water samples (e.g., from Enceladus and Europa) by the OxR instrument, because O_2_^•−^ dismutates to H_2_O_2_ and O_2_ under aqueous conditions (see reaction 1, Table 1). In such an application, the first step of the OxR instrument will record O_2_ of unknown origin (for instrument calibration) in a melted ice sample. Following this step, CAT will be administered to convert to O_2_ any present H_2_O_2_. This will be the only peroxidant specifically determined by the OxR instrument in the (melted) ice samples from the surface or plums of Enceladus and Europa.

## 6. Implementation of the OxR Instrument

**Figure 3:**
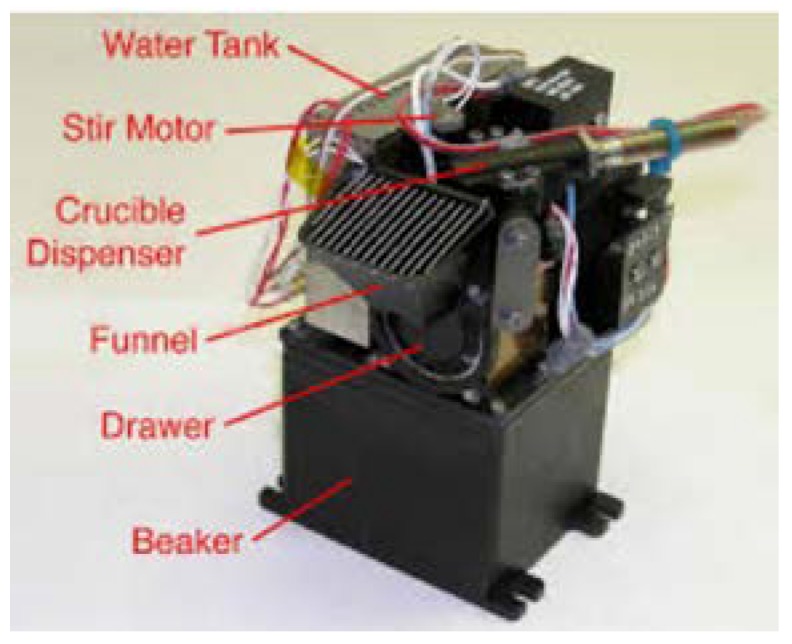
Image of the Wet Chemistry Laboratory (WCL) from the Phoenix Lander.

One approach for implementing the OxR assay for field instrument construction is to keep it compatible with the Wet Chemistry Laboratory (WCL) that flew as part of the Phoenix lander mission to Mars [30]. The WCL (Figure 3) consists of a lower beaker containing sensors designed to analyze the chemical properties of the regolith and an upper actuator assembly for adding regolith, water, reagents, and stirring [63]. The WCL sensor set included an O_2_ electrode, pressure sensor, and thermocouple needed for the OxR assay. A key part of the WCL system is the storage of liquid and dry reagents. Our prototype design uses a reagent dispenser assembly similar to WCL (which uses five crucibles to store the reagents to be dispensed). We will use the following three crucibles:

A crucible for dispensing into the beaker the anhydrous ACN to wash out from regolith any background O_2_ (for recording its level).

A crucible divided into two compartments to store the SOD enzyme and its solvent (for recording O_2_ released from the dismutation of regolith superoxide radicals; H_2_O_2_ will also be released by this dismutation) separately.

A crucible divided into two compartments to store the CAT enzyme and its solvent (for recording O_2_ released from superoxide radical-derived H_2_O_2_, and that derived from regolith peroxides) separately.

**Figure 4:**
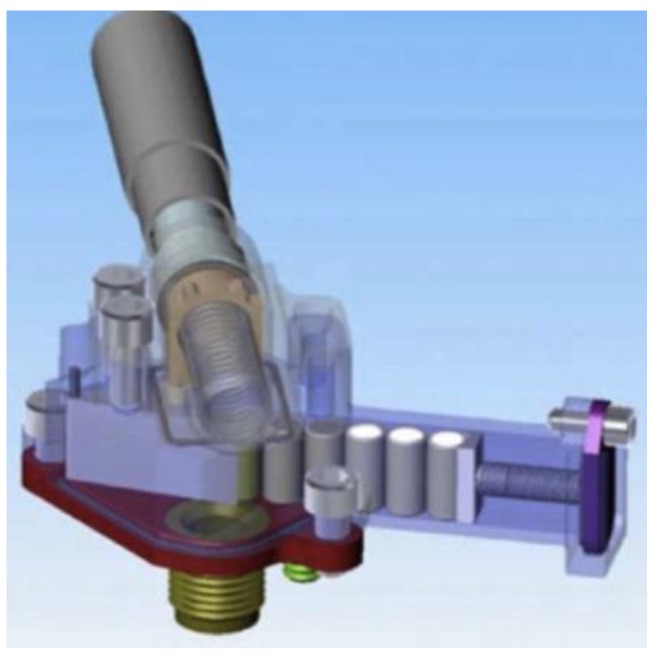
Diagram of the reagent dispenser assembly with crucibles ready for deployment [63].

**Figure 5:**
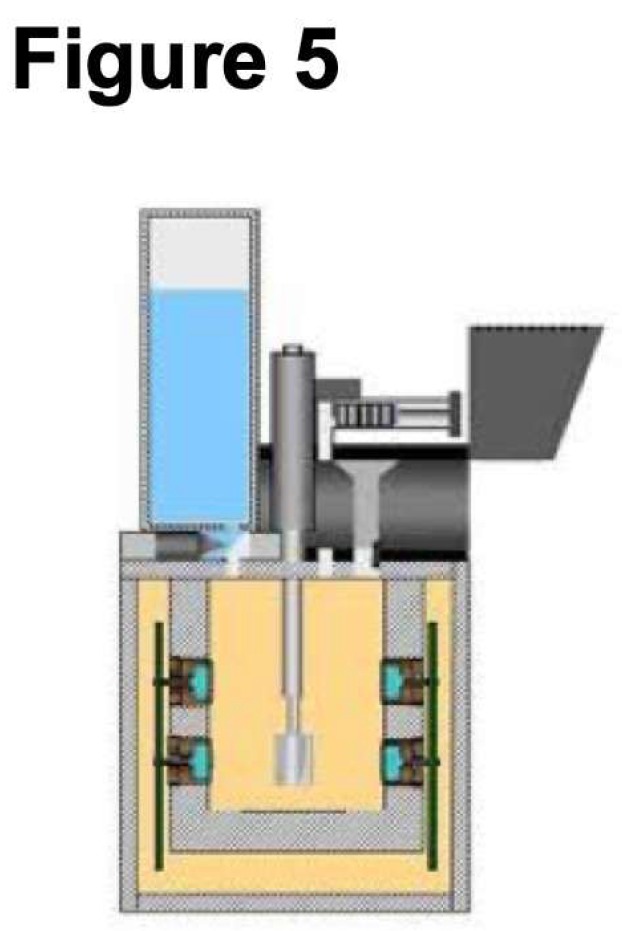
Diagram of the WCL reaction cell showing water storage and stirring rod.

**Figure 6:**
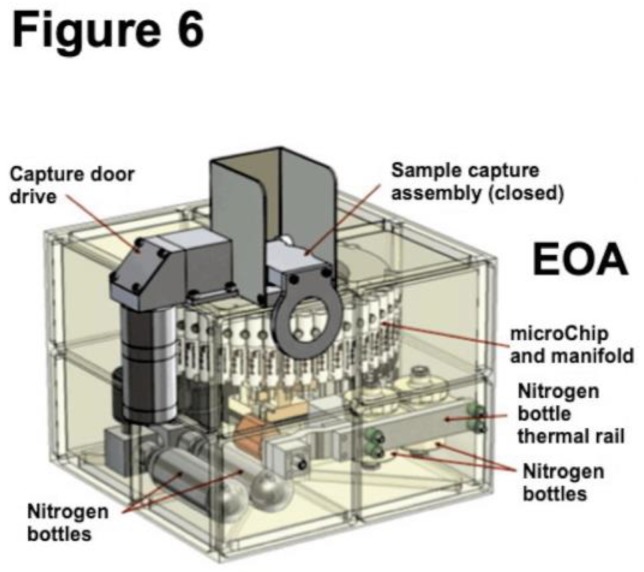
Diagram of the Enceladus Organic Analyzer (EOA) data programmable chip (modified from [65]).

The automated and sequential dispensing of the reagents is critical to the success of the prototype. A diagram of the Phoenix system is shown in Figure 4. The reagent dispenser assembly will be coupled to the construction and operational testing of the beaker (reaction cell). A diagram of the WCL reaction cell is shown in Figure 5. In contrast to the complex array of sensors in the WCL on Phoenix [63], we have only O_2_ sensors, temperature, and pressure. Joining the reagent dispenser assembly and the reaction cell completes the prototype. However, the WCL instrument uses a 25-cc chamber to analyze 1 cc of regolith. For some missions, this is an important issue and motivates microfluidics approaches. An alternative instrument construction approach will be based on the microfluidic transport/delivery technology [64,65,66,67,68,69], already developed by the R.A. Mathies’s Space Sciences Laboratory at Berkeley University. The chip for microscopic fluid transport between the components of an instrument such as OxR (e.g., reagent storage capsules, regolith sample chamber with O_2_/temp/pressure sensors, and waste reservoirs), is analogous to digital electronic processors, and all that is needed is a change in the order of operations conducted by the device [66]. All macroscopic reagent volumes are contained within stainless steel bellows expanded or contracted by externally applied N_2_ gas. The OxR instrument chip can be constructed as a scaled-down version (e.g., a 200 gr, 2 Watts, 10x10x10 cm package) of the Enceladus Organic Analyzer (EOA) chip (Figure 6).

The OxR instrument prototype will be tested in a laboratory setting and results compared to standard laboratory procedures, followed by field testing in the Mojave Desert. This has been a continuing test site for our studies [1], and, thus, we have a deep knowledge base of the site and the expected results providing a convenient basis for prototype testing.

## 7. Studies with the OxR Instrument

The OxR instrument can have the following potential applications:

Identification of the ROS O_2_^•−^ and O_2_^2−^, on the Moon and Mars, with extension to future missions to Jupiter’s satellite Europa and Saturn’s Enceladus and Titan.

Monitor the levels of ROS for astronaut health and safety, given that O_2_^•−^ can become biotoxic (via conversion of Fe^3+^/Cu^2+^ to Fe^2+^/Cu^+^, which, via the Fenton-reaction with the other ROS O_2_^•−^, will generate the highly biotoxic free radical ^•^OH [44]). Moreover, measuring dust/silica-induced ROS generation is crucial for the evaluation of possible health hazards [18] in future manned missions to Mars and Moon.

Identify mineral deposits rich in ROS to be used as an O_2g_ source for human consumption. O_2g_ can be easily produced on a large scale due to the following ROS reactions: O_2_^•−^ is converted to O_2g_ by mixing with (i) H_2_O (also releasing H_2_O_2_), or (ii) Fe^3+^ or Cu^2+^ [44]. O_2g_ can be produced from O_2_^2−^ (e.g., H_2_O_2_ also released from reaction (i)) by mixing with MnO_2_ [56] or silver, platinum, lead, ruthenate, or RuO_2_ [57].

Identify O_2_^•−^/O_2_^2−^ on the metal parts of manned space vehicles/stations. ROS can be generated by a combination of O_2g_ (in vehicle) with cosmic radiation [1]. O_2_^•−^ and O_2_^2−^ have implications for exploration because they can:

(i) cause corrosive oxidative deterioration of space vehicles/stations,

(ii) pose a serious risk for oxidative modification of stored foods, making them unsafe for astronauts,

(iii) compromise astronaut health due to their well-known biotoxic effects [44].

Identify locations on Mars and the Moon with low ROS levels which may be indicative of the high potential for biosignature (e.g., [70,71]) preservation. Of particular upcoming interest is the Dragonfly mission to Titan by NASA (launched in 2026, and landing in 2034), which will search for evidence of prebiotic chemical processes on the surface of Titan [72].

The instrument is also applicable to terrestrial research, with indicative studies being: (i) O_2_^•−^/O_2_^−^ association to microorganisms’ oxidative stress in extreme desert environments, with extension to life’s origin [1,73]; (ii) health hazard implications from measuring ROS-reactivity of (a) volcanic ash (due to ^•^OH generation) [74], and (b) pyrites (from O_2_/H_2_O_2_/surface-bound ferric iron-induced ^•^OH generation during pyrite oxidation) in coal mining regions [75].

## 8. Conclusions

We have developed a sensitive assay for the use in a future Oxygen Release (OxR) instrument for the detection of ROS, with potential applications to the Mars, Moon, Europa, Titan, and Enceladus missions. The instrument can support the exploration of the Moon (including monitoring of astronaut health hazards), exploration on Mars, Moon, and Titan, and terrestrial studies. The OxR instrument is based on the selective and specific enzymatic decomposition of supero/peroxidants to O_2_ and their quantification by the measurement of the released O_2_. An alternative non-enzymatic option is also proposed. Laboratory simulations and the sensitivity of the commercially available O_2_ sensors indicate that the OxR instrument can detect metal O_2_^•−^/O_2_^2−^ in the Martian and Lunar regolith and also O_2_^2−^ in the icy waters of the satellites of Saturn Enceladus and Titan (in its regolith too) and of Jupiter’s Europa, at levels as low as 10 ppb. In terms of Technology Readiness Level, the OxR instrument is at 3 (method validated in the lab), and can be made flight-ready by leveraging the Phoenix Wet Chemical Laboratory hardware or a microfluidic transport/delivery technology.

## Figures and Tables

**Table 1:** Aqueous decomposition of metal salts of O_2_^•−^ and O_2_^2−^

Metal (Me) Salts of O_2_^•^^−^ Release O_2_ (↑) and H_2_O_2_ by the Following Reactions [37,44]		
Adsorbed O_2_^•−^	2 O_2_^•−^_ads_ + 2 H_2_O → 2 OH^−^ + H_2_O_2_ + O_2_↑	(1)
Metal salts of O_2_^•−^	2 Me^+^ O_2_^•−^ + 2 H_2_O → 2 Me^+^OH^−^ + H_2_O_2_ + O_2_↑	(2)
Metal-O_2_^•−^ complexes	2 Me^n+^− O_2_^•−^ + 2 H_2_O → 2 Me^n+^ + 2 OH^−^ + H_2_O_2_ + O_2_↑	(3)
Metal Peroxides/Hydroperoxides Release H_2_O_2_ by the Following Reactions [37]		
Me-salts of O_2_^2−^	Me^+^_2_O_2_^2−^ + 2 H_2_O → 2 Me^+^OH^−^ + H_2_O_2_	(4)
	Me^2+^O_2_^2−^ + 2 H_2_O → Me^2+^(OH^−^)_2_ + H_2_O_2_	(5)
Me-hydroperoxides (MeO_2_H)	MeOOH + H_2_O → MeOH + H_2_O_2_	(6)

**Table 2:** Reactions of the OxR (Oxygen Release) assay.

I. Metal O_2_^•−^ (O_2_^•−^_ads_, Me^+^ O_2_^•−^, Me^n+^− O_2_^•−^); additional details for their hydrolysis/dismutation are presented in reactions 1–3, and in Figure 1 *step a*.	Step 1. Metal O_2_^•−^ (e.g., Me^+^ O_2_^•−^) dissociation reaction: Me^+^ O_2_^•−^ (in H_2_O) → O_2_^•−^ + Me^+^ *Note*: Stock solution of stable O_2_^•−^ is obtained by dissociation of Me^+^ O_2_^•−^ (e.g., KO_2_) in anhydrous acetonitrile (ACN). Step 2. Release of O_2_ (and H_2_O_2_) via SOD-catalyzed dismutation of O_2_^•−^ (from I, step 1): 2 O_2_^•−^ + 2 H_2_O → 2 OH^−^ + H_2_O_2_ + O_2_ ↑(same as reaction 1) *Note*: The spontaneous dismutation of O_2_^•−^ by H_2_O has a rate constant ~2x10^5^ M^-1^ s^-1^, while that with SOD is 32,000-fold faster; 6.4x10^9^ M^-1^ s^-1^ [52]. Step 3. Base (MeOH) formation: Me^+^ + OH^−^ → MeOH	
II. Metal O_2_^2−^ (Me^+^_2_O_2_^2−^, Me^2+^O_2_^2−^, MeOOH); additional details for their hydrolysis are presented in reactions 4–6.	Step 1. Dissociation reaction of metal O_2_^2−^ (e.g., Me^+^_2_O_2_^2^_¯_): Me^+^_2_O_2_^2−^ (in H_2_O) → O_2_^2^^−^ + 2 Me^+^ Step 2. Hydrolysis reaction of O_2_^2^^−^ (from II, step 1): O_2_^2^^−^ + 2 H_2_O → 2 OH^−^ + H_2_O_2_ (same as reaction 4) Step 3. Base (MeOH) formation: 2 Me^2+^ + 2 OH^−^ → 2 MeOH	
III. H_2_O_2_ released by the hydrolysis of metal O_2_^•−^ and O_2_^2−^; additional details are shown in Figure 1 *step b*.	Release of O_2_ via CAT-catalyzed decomposition of H_2_O_2_ [44], resulting from I, step 2, and/or II, step 2:	
	2 H_2_O_2_ → 2 H_2_O + O_2_↑	(7)
